# Multi-Resolution Image Segmentation Based on a Cascaded U-ADenseNet for the Liver and Tumors

**DOI:** 10.3390/jpm11101044

**Published:** 2021-10-19

**Authors:** Yan Zhu, Aihong Yu, Huan Rong, Dongqing Wang, Yuqing Song, Zhe Liu, Victor S. Sheng

**Affiliations:** 1 Department of Radiology, Affiliated Hospital of Jiangsu University, Zhenjiang 212001, China; salary_hi@126.com (Y.Z.); wangdongqing71@163.com (D.W.); 2 School of Computer Science and Communications Engineering, Jiangsu University, Zhenjiang 212013, China; 2221908059@stmail.ujs.edu.cn (A.Y.); yqsong@ujs.edu.cn (Y.S.); 3 School of Artificial Intelligence, Nanjing University of Information Science and Technology, Nanjing 210044, China; ronghuan@nuist.edu.cn; 4 Department of Computer Science, Texas Tech University, Lubbock, TX 79409, USA

**Keywords:** CT images, convolutional neural network, channel attention, cascaded, liver segmentation

## Abstract

The liver is an irreplaceable organ in the human body, maintaining life activities and metabolism. Malignant tumors of the liver have a high mortality rate at present. Computer-aided segmentation of the liver and tumors has significant effects on clinical diagnosis and treatment. There are still many challenges in the segmentation of the liver and liver tumors simultaneously, such as, on the one hand, that convolutional kernels with fixed geometric structures do not match complex, irregularly shaped targets; on the other, pooling during convolution results in a loss of spatial contextual information of images. In this work, we designed a cascaded U-ADenseNet with coarse-to-fine processing for addressing the above issues of fully automatic segmentation. This work contributes multi-resolution input images and multi-layered channel attention combined with atrous spatial pyramid pooling densely connected in the fine segmentation. The proposed model was evaluated by a public dataset of the Liver Tumor Segmentation Challenge (LiTS). Our approach attained competitive liver and tumor segmentation scores that exceeded other methods across a wide range of metrics.

## 1. Introduction

Since the liver is a reasonably significant organ for abdominal metabolism, liver tumors in particular, malignancy certainly poses a serious threat to human health. Statistics from the World Health Organization have also shown that commonly occurring liver cancer was accompanied by a high mortality rate worldwide. How to accurately identify, locate and segment lesions has become the primary step in the development of subsequent precision treatment and individualized protocols. Accurate diagnostic treatment has a significant positive effect on reducing the number of patients suffering from diseases and improving disease prognosis [1]. The computed tomography (CT) image analysis technique is the main solution for diagnosis, and plays a vital role in the treatment of hepatoma [2]. Precise description of the area of the liver and the lesions on CT images allows for better assessment of liver function and for producing more appropriate surgical plans [3]. Traditional manual segmentation is tedious and time-consuming. In terms of time and efficiency, semi-automatic segmentation is obviously superior to manual segmentation. Semi-automatic segmentation algorithms of CT images mainly rely on information distribution and often use model-driven algorithms such as region growth [4], thresholding [5], active contour model [6], graph cut [7], shape statistical model [8], etc. Although these image segmentation approaches can speed up the time-consuming manual segmentation process, it still suffers from over-reliance on prior knowledge. Recently, great advances have facilitated the development of typical image processing tasks in computer vision, such as image classification [9], object detection [10], image segmentation [11], etc. Fully automatic segmentation with powerful self-learning capabilities stands out in medical image analysis. This method can assist clinicians to perform follow-up analysis of lesions rapidly and stably based on the morphological characteristics of lesions with maximum fidelity, providing an objective basis for the precise formulation of lesion treatment plans [12].

Medical image segmentation algorithms based on deep-learning convolutional neural networks are especially representative in fully automated segmentation of the liver and liver tumors. Long et al. [13] from Berkeley proposed a fully convolution neural network (FCN) for the semantic segmentation of images, which extends image-level classification to pixel-level classification. A FCN replaces the fully connected layers of VGG [14] with deconvolution layers for upsampling, so that the feature maps can be returned to their original size. Sun et al. [15] applied a fully convolutional network (FCN) to CT images for segmentation of the liver and the accuracy exceeded that of numerous semi-automatic segmentation methods. Chlebus et al. [16] improved an FCN and utilized a conditional random field (CRF) in post-processing for the refinement of segmentation. Ben-Cohen et al. [17] have trained an FCN by adding weight parameters to the loss function, allowing the model to better focus on the target region of liver segmentation. Badrinarayanan et al. [18] proposed an encoder–decoder structure network. This method addressed the lack of response information in upsampling by using pooling indices. Almotairi et al. [19] modified the SegNet for efficient segmentation of the liver and lesions. Ronneberger et al. [20] presented U-Net by combining an FCN with an encoder–decoder structure. U-Net adds a skip connection structure that can fuse the low-level features together with the high-level features. Comparatively excellent segmentation results were achieved in the ISBI Cell Tracking Challenge via the model. Seo et al. [21] added residual modules with de-convolution layers and activated U-Net to improve the accuracy of segmentation.

However, the locations and shapes of the liver and liver lesions are highly variable in the different CT images [22]. It is worth mentioning that most malignant tumors of the liver are caused by the presence of underlying liver lesions, which result in variable liver morphology and irregular morphology of liver lesions. Thus, some researchers have made use of cascaded structures to form coarse-to-fine segmentation patterns. Christ et al. [22] used two FCN-8s in a cascade to first obtain the region of interest (ROI) of the liver and then to segment liver lesions in the ROI, which improved the accuracy compared to the direct method. Bi et al. [23] utilized ResNet [24] in a multiscale fusion cascade to infer boundaries of the liver and tumors. Kaluva et al. [25] applied a cascaded DenseNet [26] for to independently segment the liver and tumors to improve the precision. Segmentation of the liver and tumors still has multiple challenges at present, such as a low contrast between the organs and the high complexity of target shapes [26]. Rundo et al. [27] applied squeeze-and-excitation blocks concerning the channel attention. Jin et al. [28] used multiple attention hybrid connection blocks, combining soft and hard attention mechanisms together with long and short jump connections. Lu et al. [29] designed two stages of liver localization and tumor segmentation. The superficial spatial information is first used to improve liver identification, and the 2D image features and 3D spatial features of CT image slices are used to accurately identify liver tumors. They used attentional mechanisms to improve the segmentation performance of small liver tumors. In general, the ensemble in the convolution process results in the loss of spatial background information of the image, and convolutional kernels with fixed geometry cannot match complex, irregularly shaped targets.

In this work, we propose a cascaded U-ADenseNet network to segment CT images for the liver and its lesions. The network is designed to reduce the difficulty of segmenting areas concerning targets that involve diverse locations and complex shapes. So as to minimize the loss of spatial information during down sampling from feature extraction, U-ADenseNet replaces down sampling with dilated convolution. We innovate a multi-layered channel attention module, subtly combining atrous spatial pyramid pooling. The structure uses U-Net to initially extract the feature information of the liver and tumors, cascading our designed ADenseNet for fine segmentation afterward. The model makes use of densely connected modules to realize the feature interaction of multiple receptive fields, effectively unifying global spatial information with local depth semantic information. Our experiments applied the dataset from the Liver Tumor Segmentation Challenge competition (LiTS) [30]. Experimental results showed the improvement of the Dice similarity coefficient, volumetric overlap error and relative volume difference compared to the baseline methods, which verified the efficiency of this model.

## 2. Related Theories

### 2.1. U-Net

Earlier convolutional layers tend to learn lower-level concepts more, while the later convolutional layers produce higher-level feature maps. The number of feature maps needs to be increased when the network reaches deeper levels. A simple way to build the neural network architecture for this task is to simply stack many convolutional layers and output the final segmentation mappings. Corresponding segmentation mappings are learned from the input images by direct, successive transformations of the feature maps [31]. Therefore, the researchers often compressed the spatial resolution to moderate the computational pressure. U-Net can use a small amount of data to learn a model that is robust to edge extraction. More specifically, the U-Net architecture consists of a contracting path for capturing content and an expanding path for precise localization. The contracting path still uses the convolutional pooling component of a traditional convolutional neural network, in which channels become twice as large after a down sampling process. The expanding path consists of a 2 × 2 deconvolution, where output channels of the deconvolution machine are half of the original number of channels and then concatenated with the original feature map to obtain a feature map with the same number of channels as the original, followed by two convolutions of size 3 × 3 and the action of Relu. Cropping the feature map is necessary because there is a loss of boundary pixels during the convolution process. The desired target species are obtained in the last layer by the action of convolution with a convolution kernel size of 1 × 1. There are 23 convolutional layers in U-Net. However, this network needs to choose the size of the input images carefully to ensure that all max pooling operations act on feature maps with even length and width. This architecture has already been commonly used in medical segmentation tasks.

### 2.2. DenseNet

Convolutional networks can be significantly deeper, more accurate and easier to train if they contain shorter connections between the layers closer to the input and the layers closer to the output. The traditional structure is delivered from layer to layer, and some information is changed whilst some information is retained in the delivered information. DenseNet starts with features and achieves better results and fewer parameters by making the best use of features. DenseNet improves the efficiency of information and gradient transfer in the network. Each layer obtains the gradient directly from the loss function and obtains the input signal directly, so that a deeper network can be trained, and this structure also has the effect of regularization [26]. This connection allows for a more efficient transfer of features and gradients. Other networks are dedicated to improving the network performance from depth and width, but DenseNet is dedicated to improving the network performance from the feature reuse perspective. It creates short paths from early layers to later layers.

### 2.3. Atrous Spatial Pyramid Pooling

The benefit of down sampling the feature mapping is the extended perceptual field for the fixed, constant convolutional kernel size. The approach makes more sense than increasing the size of the convolutional kernel because of the lower parametric efficiency of large-size convolutional kernels. However, this expansion is involved in the reduction in spatial resolution. Dilated convolution provides an alternative method to obtain a wide field of view while preserving the full spatial dimensionality. Dilated convolution separates the space with values based on a specified dilation rate. The pooling layers are replaced by dilation convolutions with continuously increasing dilation rates, which can prevent the loss of spatial details while maintaining the same perceptual field [32]. Atrous spatial pyramid pooling (ASPP) is based on dilated convolution and spatial pyramid pooling. ASPP samples the given input in parallel with null convolution at different sampling rates, which is equivalent to capturing the context of the image at multiple scales. ASPP is actually a version of spatial pyramid pooling. In ASPP, parallel cavity convolution with different rates is applied in the input feature mapping and fused together. Since objects of the same class may have different scales of images, ASPP helps to consider different object scales [33].

## 3. Proposed Method

### 3.1. Cascaded U-ADenseNet Network Architecture

**Figure 1:**
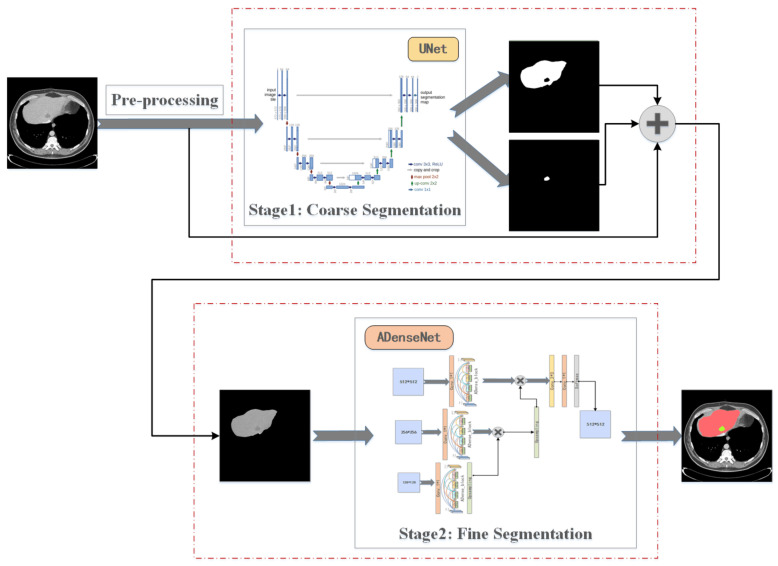
Main steps of this work for the segmentation of the liver and tumors.

In order to extract more effective features from the liver and liver tumors, this work is conducted by using the method of coarse-to-fine segmentation. Multiple network architectures are utilized to implement liver and tumor image segmentation tasks. The general steps of such a coarse-to-fine segmentation can be illustrated in Figure 1. The main steps of this work include pre-processing, coarse segmentation and fine segmentation. Pre-processing is an indispensable part of the medical image segmentation before the feature extraction. The output of the first stage is used as the input of the second stage, which combines multi-level information and extracts more image features to improve the segmentation accuracy. The first stage of coarse segmentation uses a U-shaped network structure to obtain contextual and location information, which is called U-Net. That is an initial prediction of the liver and tumor region in the image. The second stage applies our designed ADenseNet for the fine segmentation. The final results of refined segmentation of the liver and liver tumors is obtained.

In detail, pooling will lead to the excessive loss of spatial information of images. A mismatch between convolutional kernels with a fixed geometric structure and complex, irregularly shaped targets of the liver and tumors limits segmentation performance. In order to address these problems, this new network is designed with a coarse-to-fine principle and applies to the segmentation of the liver and tumors. Figure 1 shows the structure of the cascaded U-ADenseNet network proposed in this paper. It mainly consists of two major parts. After being trained by the feature extraction network in the form of U-Net, a feature enhancement extraction network in the form of ADenseNet was designed in a cascade. The probability maps of the liver and its lesions obtained from the first-stage segmentation were fused with the original map using addition. Afterward, they are entered into the second-stage segmentation network and processed to output optimized segmentation predictions. This network is designed to reduce the impact of the complex and irregular characteristics of the liver and its tumors by narrowing the region of interest (ROI) and unifying global spatial information with local depth semantic information.

### 3.2. Preprocessing

The purpose of image pre-processing is to reduce the interference of complex backgrounds on the target area. The publicly available dataset used in our experiments of this study was CT images from the Liver Tumor Segmentation Challenge (LiTS) [30]. These CT slices contained two foreground classes (i.e., liver and liver tumors) and a background class consisting of unrelated organs. The initial effects of image enhancement and noise reduction were achieved by performing mainly *HU* windowing and Gaussian filtering on the images. The formula for *HU* windowing is shown below:

(1) HU=pixel∗slope+intercept

These images have different slope and intercept values. The parts we want are filtered according to HU, and all other parts are blacked out or whitened. This reduces the interference of the complex background on the target areas. In addition, Gaussian filtering is a linear smoothing filter that is suitable for eliminating noise. Gaussian filtering is realized by a discretization window and sliding window convolution with a Gaussian template.

### 3.3. ADenseNet

#### 3.3.1. ADenseNet Network Architecture

**Figure 2:**
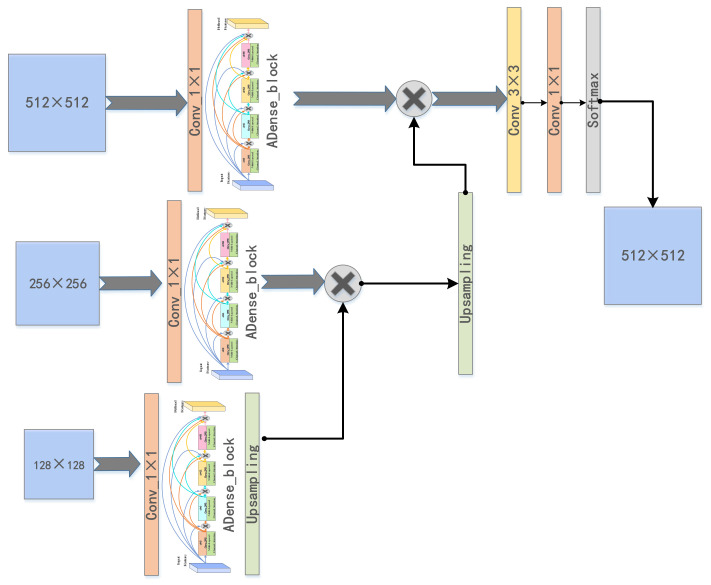
The specified structure of the ADenseNet network.

**Figure 3:**
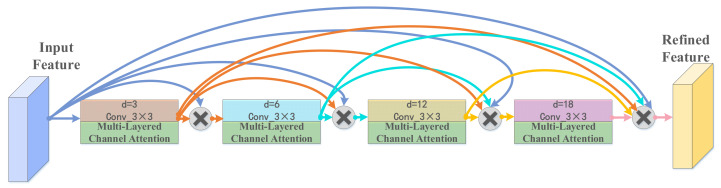
The detailed technical principles of an ADense block.

The information on multiple image resolutions is critical to capturing target location details, so the interactions between multiple image resolutions need to be learned to obtain more feature information. The second stage of the cascaded U-ADenseNet network in the study of the segmentation of the liver and its tumors is demonstrated in Figure 2. The coarse segmentation prediction images were obtained after the first stage of U-Net. These images fused original, pretreated CT slices to acquire the coarse positioning of CT images. The resolution of these coarse positioning images was 512 × 512, and the data were scaled down to 256 × 256 and 128 × 128 when expanded. Convolution and ADense block operation were carried out after the input of CT images with three different resolutions, followed by bi-linear difference sampling and feature image fusion. Finally, convolution and softmax operations were implemented to output the predicted segmentation results. Our approach captures the interactions between the multiple image resolutions simultaneously in a fully learned end-to-end optimization. This work involves the ADense block in the ADenseNet for fine segmentation of the liver and tumors. Figure 3 shows the technical principles of the ADense block. In general, long-distance contextual information and information at different scales were important for results.

In order to increase the receptive field, the extracted feature map is often pooled to achieve the effect of increasing the receptive field, combined with the multi-scale information by jumping connections. Since pooling is conducted in a direct and crude way, the spatial resolution will be sacrificed after each pooling, leading to the loss of spatial information after multiple pooling, even doing harm to the segmentation effectiveness. The emergence of dilated convolution is to solve the problem of enhancing the receiver field without a loss of information. Atrous spatial pyramid pooling will parallel or cascade the convolution of different dilation rates to obtain multi-scale information gain. Due to its mechanism of dilated convolution, only a small number of pixels are selected for each calculation and the sampling is not intensive, resulting in a large amount of discarded spatial information. Therefore, Adense_block applies the ideology of dense connection to atrous spatial pyramid pooling.

#### 3.3.2. Multi-Layered Channel Attention

**Figure 4:**
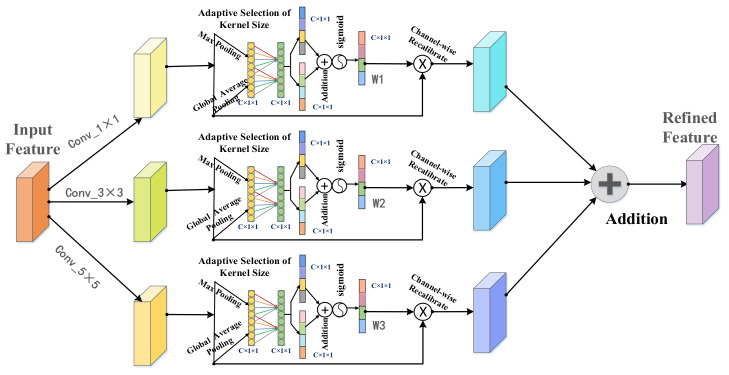
Mechanism of the multi-layered channel attention.

For the purpose of reducing the loss of important features of the CT images in the training process and causing segmentation results to be more accurate, it must enhance the features of complex segmentation targets via multi-layered channel attention. The design of multi-layered channel attention is aimed at reducing the difficulty of segmenting the complex liver and tumors, by selecting information of different spatial scales across channels. Figure 4 demonstrates the whole process of multi-layered channel attention. Feature maps, after three kinds of convolutions, respectively formed three branches. Each branch carries out the maximum pool and global average pool operation, then shared a network layer to obtain the weight of the channel feature. In detail, we changed the feature map from C × H × W to C × 1 × 1 through the global average pooling and max pooling method, and we used K × 1 × 1 convolution to process information and obtain a C-dimensional vector. We used the sigmoid function to normalize data and obtained the corresponding weights. Finally, through channel-wise multiplication, the feature map after information calibration is obtained. The feature maps of the original branch channel feature calibration; finally it will be treated as a three-branch operation after processing additive operation feature maps, obtaining more accurate features. In conclusion, this multi-layered channel attention mechanism can focus the updating direction of the weights in the model to the information which was helpful to the segmentation tasks.

### 3.4. Cross-Entropy Adaptive Weight Loss Function

Since this article has dealt with the task of multi-class segmentation of the liver and liver tumors, the loss function of adaptive, weighted cross-entropy was used. The error value of the loss function was calculated at the endpoint of the forward propagation, and the loss layer was also the starting point of the back propagation. Different weights can be assigned to the different categories when the unbalanced categories were in the samples. Cross-entropy measures the degree of difference between three different probability distributions for the same random variable, and can be expressed in machine learning as the difference between the true probability distribution and the predicted probability distribution. The smaller the value of cross-entropy, the better the model prediction. The formula of the adaptive weighting method was as follows:

(2) $$L\left(y,\hat{y}\;\right)=-\frac{1}{N}\sum_{i=1}^{N}\sum_{c=1}^{3}w_{i}^{c}y_{i}^{c}log{\hat{y}}_{i}^{c},$$

Among the formula, $y_{i}^{c}$ means the probability that an image, *i*, falls into category, *c,* which included background, liver or liver tumors. $w_{i}^{c}$ stands for weight, ${\hat{y}}_{i}^{c}$ represents the truth tag for an image, *i*. Moreover, the weighting of the categories was defined by the following formula:

(3) $$\omega_{class}=\frac{1}{\ln\left(t+P_{class}\right)},$$

Here, $P_{class}$ can be seen as the proportion of the sample of that class. Additionally, we set *t*, which was a hyper-parameter, to 1.02. Then, we limited $\omega_{class}$ to (1.0, 50). The implementation of weighted cross-entropy is to add weights to the different categories so that the network gives importance to the categories with smaller sample sizes.

## 4. Experimental Results

### 4.1. Dataset and Implementation Details

We adopted Pytorch to implement this model for medical CT images segmentation of the liver and tumors through running the Linux Ubuntu 16.04 64-bit operating system. Our experimental instrument was equipped with an NVIDIA RTX 2080 Ti 11 GB GPU on an Intel Core i7-7700K 4.20 GHz with 16 GB RAM. The cascaded U-ADenseNet network training performed by 300 epochs on a single GPU with a constant initialized learning rate was 0.0001 and the initialized alpha was 0.33.

**Figure 5:**
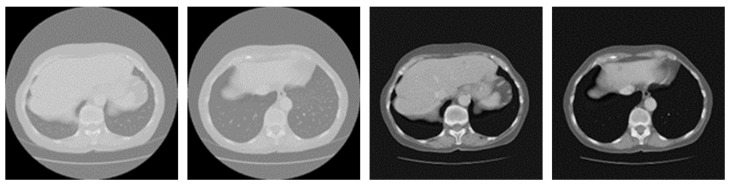
Comparison of the original images and the processed images.

The LiTS dataset contained 131 cases of enhanced abdominal CT scans with labels in the formal way, originally in a neuroimaging informatics technology initiative (NIFTI) format. Because these CT volumes were acquired from multiple clinical sites by different scanners and protocols, the images varied widely in their appearance. The data were divided into a training set of 105 cases and a testing set of 26 cases, which were processed into axial slices in a portable network graphics (PNG) format. Each CT slice has a resolution of 512 × 512. The all-black images that did not contain the liver and tumors were removed from the training set, speeding up the training process for the liver and tumors in order to reduce the training burden. Considering the problems of blurred boundaries and low contrast between the targets and other surrounding organs in the medical images of the LiTS dataset, the images needed to be processed to reduce the interference of extraneous noise and enhance the contrast. Figure 5 illustrates the typical comparison of the original images and the processed images. Due to the special nature of medical CT images, it can be seen that the details of the edges of the organs and the contrast of the images were more distinct after data preprocessing. In the experiment, these processed CT images were fed into the cascaded U-ADenseNet, and then the network produced the predicted results of segmentation concerning the liver and tumors.

### 4.2. Performance Metrics

To validate and measure the accuracy of predicted results and the effectiveness of the segmentation algorithm, this section used three evaluation metrics to validate the segmentation results of the liver and liver tumors. The Dice similarity coefficient (DSC) can be seen as the most common evaluation metric in the field of segmentation algorithms. The Dice similarity coefficient can be calculated by the following formula:

(4) $$\mathrm{DSC}=\frac{2\left|A\;+\;B\right|}{\left|A\right|+\left|B\right|},$$

Here, A denotes the predicted segmentation result and B indicates the ground truth. Furthermore, volumetric overlap error (VOE) and relative volume difference (RVD) were used as evaluation metrics. VOE represents the overlap between the segmentation result and the actual segmentation result. RVD indicates the difference between the splitting results and the volume between markers. The specific calculation formulas are as follows:

(5) $$\mathrm{VOE}=1-\frac{\mathrm{TP}}{\mathrm{TP}\;+\;\mathrm{FP}\;+\;\mathrm{FN}},$$

(6) $$\mathrm{RVD}=\frac{\mathrm{FP}}{\mathrm{TP}\;+\;\mathrm{FN}}\;,$$

Here, TP, FP and FN denote the number of pixels that are predicted with the correct foreground label (true positive), the number of background pixels that are erroneously predicted as foreground pixels (false positive) and the number of foreground pixels that are erroneously predicted as background pixels.

### 4.3. Experimental Results and Analysis

#### 4.3.1. General Comparison on Ablation Experiments

To verify the effectiveness of the algorithm concerning the simultaneous segmentation of the liver and liver tumors in this paper, we implemented several experiments. The ablation results are shown in Table 1. The core idea of a cascading U-Net is to use the output of the previous network as the input of the next network, combining multi-level information and extracting more sets of image features to improve segmentation accuracy. Densely-CNN used DenseNet to independently train segmentation models of the liver and tumor in addition to precisely locate the liver region in the CT images to assist the tumor model, thus producing a joint segmentation of the liver and tumors as a way to improve segmentation accuracy. ADenseNet1 in Table 1 represents the second stage of fine segmentation merely using multi-resolution inputs without the attention mechanism. ADenseNet2 denotes a model using the attention mechanism, without the adoption of a multi-resolution input pattern during the second stage. ADenseNet1 was similar in structure to the second stage of Densely-CNN. The outstanding advantage of DenseNet was that it can encourage feature reuse. Experimental results of the U-Net-cascaded ADenseNet2 will prove the necessity to use attention mechanisms of segmentation.

We propose the model of cascaded U-ADenseNet for the coarse-to-fine segmentation with the addition of dilated convolution and a channel attention mechanism. The goal is to increase the receptive field and reduce the loss of spatial resolution information. We implemented the ablation experiments to study the effect of different technical points of this cascade network on the performance of the segmentation network. Specifically, Table 1 shows the evaluation scores of experiments. Cascaded U-ADenseNet means that the network contains the technologies mentioned above.

#### 4.3.2. Analysis of Results in Three Patterns

We have implemented experiments for different patterns of liver tumors. Pattern one refers to predicting large liver tumors, pattern two represents the prediction of small liver tumors and pattern three indicates predicting multiple liver tumors.

**Figure 6:**
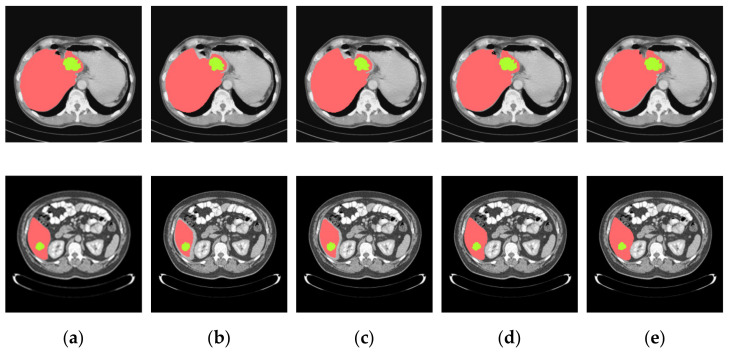
Segmentation results for the liver and tumors of pattern one. (**a**) Ground truths, (**b**) U-Net, (**c**) U-Net + ADenseNet1, (**d**) U-Net + ADenseNet2 and (**e**) cascaded U-ADenseNet.

**Figure 7:**
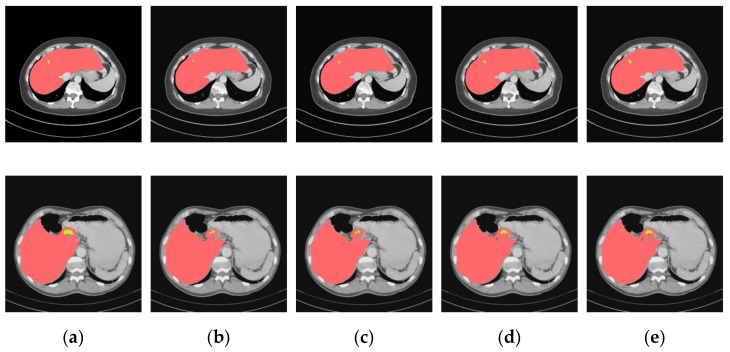
Segmentation results for the liver and tumors of pattern two. (**a**) Ground truths, (**b**) U-Net, (**c**) U-Net + ADenseNet1, (**d**) U-Net + ADenseNet2 and (**e**) cascaded U-ADenseNet.

**Figure 8:**
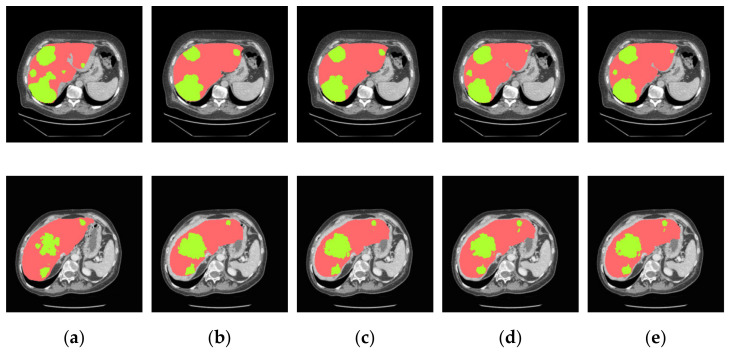
Segmentation results for the liver and tumors of pattern three. (**a**) Ground truths, (**b**) U-Net, (**c**) U-Net + ADenseNet1, (**d**) U-Net + ADenseNet2 and (**e**) cascaded U-ADenseNet.

In this paper, a pixel ratio of liver-to-tumor greater than 10% is defined as a large tumor and one less than 10% is defined as a small tumor. The threshold value is selected according to the pixel ratio of liver-to-tumor, which can be adjusted according to specific, actual scenes. Some typical examples of ground truths and the segmented predicted images of the training model for several cases are shown in the following figures. These cases refer to those from Table 1 and they are arranged in five columns from left to right in Figure 6, Figure 7 and Figure 8. The red parts refer to the liver regions and the green parts denote the region of the liver tumors. At first, from Figure 6 we can analyze that the segmentation results using the cascaded U-ADenseNet are closer than others to the contours of the ground truths when the morphologies of liver tumors are large. The main change is in the way of data input. Learning of convolutional networks is usually based on a single, fixed-resolution image. However, according to some studies, this single resolution may not be optimal and depends on the size of the objects in the image. Information from multiple image resolutions may be crucial to capture details, especially in the field of medical images. It obtains more feature information by learning the interactions between multiple image resolutions of the same image.

Moreover, Figure 7 demonstrates the segmentation results of applying these different networks in pattern two, which refers to the segmentation prediction of small liver tumors. Long-distance contextual information and information of different scales are important for segmentation results. Therefore, in order to increase the perceptual field, the extracted feature map is often pooled to achieve the effect of increasing the perceptual field, and the multi-scale information is combined by jumping connections. Since pooling is a direct and brutal way, the spatial resolution is sacrificed after each pooling, and multiple pooling may cause information loss and affect the segmentation effect. Dilation convolution emerged to solve the problem, by not losing information while improving the perceptual field. ASPP stacks nulls of different dilation rates in parallel or in a cascade to gain multi-scale information.

Furthermore, in Figure 8 we find that the predicted results of utilizing cascaded U-ADenseNet are obvious superior to others during the processing and predicting of CT images containing multiple liver tumors. Dilation convolution is not densely sampled because only a small number of pixels are selected for each calculation, and a large amount of information is discarded; when the null rate increases to a certain degree (e.g., dilation rate > 24), null convolution becomes less effective or even ineffective. Therefore, the ADense block applies the idea of dense concatenation to ASPP, and the input of each hole convolution layer is the stitching of the output and input feature map of all previous convolution layers. Therefore, the addition of multi-resolution image input and the multi-layered channel attention has significantly greater advantages.

In pattern one, the pixels of each tumor account for more of the total pixels of the liver. The difference between U-Net + ADenseNet2 and U-ADenseNet is whether the input is performed in the way of multi-resolution in the second stage, and a single-resolution input will be better than a multi-resolution input when the liver is large, so the Dice metrics using the cascaded U-ADenseNet in pattern one are slightly lower than U-Net + ADenseNet2. It can be seen clearly in Table 2 that there are higher Dice metrics in pattern two and pattern three when using the cascaded U-ADenseNet. From the evaluation of these experimental results and the comparison of segmentation images, the multi-resolution input and the attention mechanism in this cascaded network definitely play a great advantage. In particular, the multi-layered channel attention module has the most obvious effect of enhancing model performance.

#### 4.3.3. Comparison of Various Algorithms

Meanwhile, the algorithm proposed in this work was compared to other typical segmentation algorithms, such as Densely-CNN [25], USE-Net [27], RA-UNet [28] and DCU-Net [29]. It can be seen from Table 3 that the algorithm presented in this paper has obvious advantages under various evaluation metrics compared to other counterparts. DCUNet is also a two-stage segmentation and applies attention mechanisms; the implementation is related to the training of 2D slices and 3D images, and our paper uses 2D slices for training all the time. DCUNet adds the attention module to the skip connections; our method of multi-layered channel attention is added to atrous spatial pyramid pooling. In addition, the second stage of the method in this paper adopts a multi-resolution approach and atrous spatial pyramid pooling to achieve the interaction of feature information. In the experimental results, although the liver Dice of our method was not as high as for DCUNet, the tumor Dice of our method was higher than for DCUNet. This indicates that our method performs better for complex regions.

**Figure 9:**
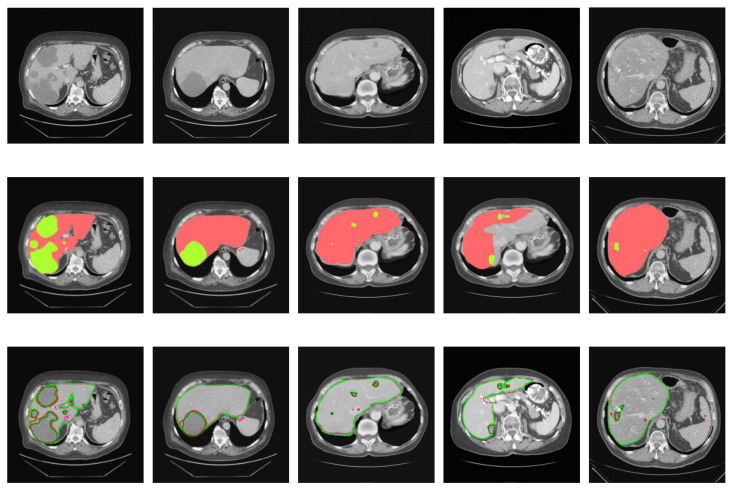
Typical segmentation results of the cascaded U-ADenseNet for the liver and tumors.

The segmentation accuracy has been improved to a certain extent, which can verify the effectiveness of the cascaded U-ADenseNet. Some examples of results, referring to the images that were predicted by the processing of our proposed network. It can be demonstrated in Figure 9.

Results of the liver segmentation contours are marked in light-green and the ground truths are marked in light-red. The predicted results of liver tumor segmentation contours are marked in dark-green and the ground truths are marked in dark-red. In Figure 9, the first row shows the original CT images before entering the network. The second row demonstrates the coloring visualization of the predicted image: the red parts represent the liver and the green parts represent the tumors. The third row displays the comparison of predicted image contours and the ground truths. These images clearly show the proposed cascaded U-ADenseNet network, whose performance is quite close to the corresponding ground truth, and few problems concerning over-segmentation and under-segmentation can be observed. This model, based on U-Net and DenseNet for coarse-to-fine segmentation of the liver and tumors, will better facilitate smart healthcare and diagnosis.

## 5. Conclusions

Fully automated medical image segmentation has become a key research technology for clinical disease diagnosis and treatment. In this study, patterns concerning the accuracy of various segmentation methods for different morphological cases of liver tumors were found. This study presented the novel U-ADenseNet network in a cascade. The techniques of dilated convolution and the multi-layered channel attention module enabled the network to extract more discriminative image features, which helps to improve the overall segmentation performance of the liver and tumors. At the same time, this two-step cascade method also solved the problem of unbalanced tumor segmentation data caused by the small proportion of liver tumors in the whole image. These experimental results on the publicly available LiTS dataset confirmed the effectiveness of the liver and tumors segmentation. For precision medicine, suitable automatic segmentation methods can be selected in the future based on the general characteristics of the liver lesions. The method of the cascaded U-ADenseNet is the most suitable one for the segmentation of multiple liver lesions, with the highest accuracy compared to some other methods. This method has some gaps in the performance of the state-of-the-art method of segmenting the liver and tumors separately, but this method has the advantages of simultaneous segmentation. Our method can be efficiently used for the segmentation of multiple lesions. Since the algorithm in the paper may cause the loss of some information during pre-processing for the given CT images, more pretreatment before feature extraction will be incorporated in future work, which can better assist the diagnosis of liver tumors together with treatment and other clinical application tasks in the liver.

## Figures and Tables

**Table 1:** Segmentation results of the liver and liver tumors on ablation analysis.

Models	Liver			Liver Tumors		
	Dice	VOE	RVD	Dice	VOE	RVD
U-Net	0.938	0.165	0.057	0.659	0.493	−0.381
U-Net + ADenseNet1	0.953	0.124	0.025	0.717	0.371	−0.193
U-Net + ADenseNet2	0.961	0.103	0.029	0.731	0.387	−0.175
Cascaded U-ADenseNet	0.963	0.086	0.023	0.745	0.353	−0.124

**Table 2:** Comparison of results in the different patterns of morphological liver tumors.

Models	Dice (Pattern One)	Dice (Pattern Two)	Dice (Pattern Three)
U-Net	0.913	0.675	0.628
U-Net + ADenseNet1	0.924	0.683	0.641
U-Net + ADenseNet2	0.931	0.711	0.663
Cascaded U-ADenseNet	0.928	0.719	0.687

**Table 3:** Segmentation performance and comparison of various algorithms.

Models	Liver			Liver Tumors		
	Dice	VOE	RVD	Dice	VOE	RVD
Densely-CNN [25]	0.923	0.015	−0.008	0.625	0.411	19.705
USE-Net [27]	0.956	0.090	0.0703	0.741	0.240	−0.190
RA-UNet [28]	0.961	0.074	0.002	0.595	0.389	−0.152
DCUNet [29]	0.967	-	-	0.725	-	-
Cascaded U-ADenseNet	0.963	0.086	0.023	0.745	0.353	−0.124

## References

[1] Seehawer M., Heinzmann F., D’Artista L., Harbig J., Roux P.-F., Hoenicke L., Dang H., Klotz S., Robinson L., Doré G. (2018). Necroptosis microenvironment directs lineage commitment in liver cancer. Nature.

[2] Azer S.A. (2019). Deep learning with convolutional neural networks for identification of liver masses and hepatocellular carcinoma: A systematic review. J. Gastrointest. Oncol..

[3] Lei T., Wang R., Zhang Y., Wan Y., Liu C., Nandi A.K. (2021). DefED-Net: Deformable Encoder-Decoder Network for Liver and Liver Tumor Segmentation. IEEE Trans. Radiat. Plasma Med. Sci..

[4] Lu X., Wu J., Ren X., Zhang B., Li Y. (2014). The study and application of the improved region growing algorithm for liver segmentation. Optik.

[5] Das A., Sabut S.K. (2016). Kernelized Fuzzy C-means Clustering with Adaptive Thresholding for Segmenting Liver Tumors. Procedia Comput. Sci..

[6] Zareei A., Karimi A. (2016). Liver segmentation with new supervised method to create initial curve for active contour. Comput. Biol. Med..

[7] Li G., Chen X., Shi F., Zhu W., Tian J., Xiang D. (2015). Automatic Liver Segmentation Based on Shape Constraints and Deformable Graph Cut in CT Images. IEEE Trans. Image Process..

[8] Tomoshige S., Oost E., Shimizu A., Watanabe H., Nawano S. (2014). A conditional statistical shape model with integrated error estimation of the conditions; Application to liver segmentation in non-contrast CT images. Med. Image Anal..

[9] Wang W., Iwamoto Y., Han X., Chen Y.-W., Chen Q., Liang D., Lin L., Hu H., Zhang Q. Classification of Focal Liver Lesions Using Deep Learning with Fine-Tuning. Proceedings of the 2018 International Conference on Digital Medicine and Image Processing.

[10] Lee S., Bae J.S., Kim H., Kim J.H., Yoon S. Liver Lesion Detection from Weakly-Labeled Multi-phase CT Volumes with a Grouped Single Shot MultiBox Detector. Proceedings of the Medical Image Computing and Computer Assisted Intervention—MICCAI 2018.

[11] Zhou Z., Rahman Siddiquee M.M., Tajbakhsh N., Liang J. UNet++: A Nested U-Net Architecture for Medical Image Segmentation. Proceedings of the Deep Learning in Medical Image Analysis and Multimodal Learning for Clinical Decision Support.

[12] Huang Q., Ding H., Wang X., Wang G. (2018). Fully automatic liver segmentation in CT images using modified graph cuts and feature detection. Comput. Biol. Med..

[13] Long J., Shelhamer E., Darrell T. Fully convolutional networks for semantic segmentation. Proceedings of the 2015 IEEE Conference on Computer Vision and Pattern Recognition (CVPR).

[14] Simonyan K., Zisserman A. (2014). Very Deep Convolutional Networks for Large-Scale Image Recognition. arXiv.

[15] Sun C., Guo S., Zhang H., Li J., Chen M., Ma S., Jin L., Liu X., Li X., Qian X. (2017). Automatic segmentation of liver tumors from multiphase contrast-enhanced CT images based on FCNs. Artif. Intell. Med..

[16] Chlebus G., Schenk A., Moltz J.H., van Ginneken B., Hahn H.K., Meine H. (2018). Automatic liver tumor segmentation in CT with fully convolutional neural networks and object-based postprocessing. Sci. Rep..

[17] Ben-Cohen A., Diamant I., Klang E., Amitai M., Greenspan H. Fully Convolutional Network for Liver Segmentation and Lesions Detection. Proceedings of the Deep Learning and Data Labeling for Medical Applications.

[18] Badrinarayanan V., Handa A., Cipolla R. (2015). SegNet: A Deep Convolutional Encoder-Decoder Architecture for Robust Semantic Pixel-Wise Labelling. arXiv.

[19] Almotairi S., Kareem G., Aouf M., Almutairi B., Salem M.A.M. (2020). Liver Tumor Segmentation in CT Scans Using Modified SegNet. Sensors.

[20] Ronneberger O., Fischer P., Brox T. U-Net: Convolutional Networks for Biomedical Image Segmentation. Proceedings of the Medical Image Computing and Computer-Assisted Intervention—MICCAI 2015.

[21] Seo H., Huang C., Bassenne M., Xiao R., Xing L. (2020). Modified U-Net (mU-Net) With Incorporation of Object-Dependent High Level Features for Improved Liver and Liver-Tumor Segmentation in CT Images. IEEE Trans. Med. Imaging.

[22] Christ P.F., Elshaer M.E.A., Ettlinger F., Tatavarty S., Bickel M., Bilic P., Rempfler M., Armbruster M., Hofmann F., D’Anastasi M. Automatic Liver and Lesion Segmentation in CT Using Cascaded Fully Convolutional Neural Networks and 3D Conditional Random Fields. Proceedings of the Medical Image Computing and Computer-Assisted Intervention—MICCAI 2016.

[23] Bi L., Kim J., Kumar A., Feng D. (2017). Automatic Liver Lesion Detection using Cascaded Deep Residual Networks. arXiv.

[24] He K., Zhang X., Ren S., Sun J. Deep Residual Learning for Image Recognition. Proceedings of the 2016 IEEE Conference on Computer Vision and Pattern Recognition (CVPR).

[25] Kaluva K.C., Khened M., Kori A., Krishnamurthi G. (2018). 2D-Densely Connected Convolution Neural Networks for automatic Liver and Tumor Segmentation. arXiv.

[26] Huang G., Liu Z., Maaten L.V.D., Weinberger K.Q. Densely Connected Convolutional Networks. Proceedings of the 2017 IEEE Conference on Computer Vision and Pattern Recognition (CVPR).

[27] Rundo L., Han C., Nagano Y., Zhang J., Hataya R., Militello C., Tangherloni A., Nobile M.S., Ferretti C., Besozzi D. (2019). USE-Net: Incorporating Squeeze-and-Excitation blocks into U-Net for prostate zonal segmentation of multi-institutional MRI datasets. Neurocomputing.

[28] Jin Q., Meng Z., Sun C., Wei L., Su R. (2020). RA-UNet: A hybrid deep attention-aware network to extract liver and tumor in CT scans. Front. Bioeng. Biotechnol..

[29] Meng L., Zhang Q., Bu S. (2021). Two-Stage Liver and Tumor Segment-ation Algorithm Based on Convolutional Neural Network. Diagnostics.

[30] Bilic P., Christ P.F., Vorontsov E., Chlebus G., Chen H., Dou Q., Fu C.-W., Han X., Heng P.-A., Hesser J. (2019). The Liver Tumor Segmentation Benchmark (LiTS). arXiv.

[31] Kayalibay B., Jensen G., van der Smagt P. (2017). CNN-based Segmentation of Medical Imaging Data. arXiv.

[32] Wei Y., Xiao H., Shi H., Jie Z., Feng J., Huang T.S. Revisiting Dilated Convolution: A Simple Approach for Weakly- and Semi-Supervised Semantic Segmentation. Proceedings of the 2018 IEEE/CVF Conference on Computer Vision and Pattern Recognition.

[33] Chen L.-C., Zhu Y., Papandreou G., Schroff F., Adam H. Encoder-Decoder with Atrous Separable Convolution for Semantic Image Segmentation. Proceedings of the Computer Vision—ECCV 2018.

